# Understanding the role of miRNAs in cervical cancer pathogenesis and therapeutic responses

**DOI:** 10.3389/fcell.2024.1397945

**Published:** 2024-08-28

**Authors:** Prashant Chauhan, Sreepoorna Pramodh, Arif Hussain, Deena Elsori, Sorabh Lakhanpal, Rahul Kumar, Mohammed Alsaweed, Danish Iqbal, Pratibha Pandey, Ayoub Al Othaim, Fahad Khan

**Affiliations:** ^1^ MPA Research, Greater Noida, Uttar Pradesh, India; ^2^ Department of Biomedical Sciences, University of Birmingham Dubai, Dubai, United Arab Emirates; ^3^ School of Life Sciences, Manipal Academy of Higher Education, Dubai, United Arab Emirates; ^4^ Faculty of Resilience, Rabdan Academy, Abu Dhabi, United Arab Emirates; ^5^ School of Pharmaceutical Sciences, Lovely Professional University, Phagwara, Punjab, India; ^6^ Chitkara Centre for Research and Development, Chitkara University, Baddi, Himachal Pradesh, India; ^7^ Department of Medical Laboratory Sciences, College of Applied Medical Sciences, Majmaah University, Al-Majmaah, Saudi Arabia; ^8^ Department of Health Information Management, College of Applied Medical Sciences, Buraydah Private Colleges, Buraydah, Saudi Arabia; ^9^ Centre for Research Impact and Outcome, Chitkara University Institute of Engineering and Technology, Chitkara University, Rajpura, Punjab, India; ^10^ Centre for Research Impact and Outcome, Chitkara College of Pharmacy, Chitkara University, Rajpura, Punjab, India; ^11^ Center for Global Health Research, Saveetha Medical College and Hospital, Saveetha Institute of Medical and Technical Sciences, Chennai, Tamil Nadu, India

**Keywords:** tumour suppressor, miRNAs, cervical cancer, oncogenic mirnas, signalling pathway

## Abstract

Cervical cancer (CC) is the most common cancer in women and poses a serious threat to health. Despite familiarity with the factors affecting its etiology, initiation, progression, treatment strategies, and even resistance to therapy, it is considered a significant problem for women. However, several factors have greatly affected the previous aspects of CC progression and treatment in recent decades. miRNAs are short non-coding RNA sequences that regulate gene expression by inhibiting translation of the target mRNA. miRNAs play a crucial role in CC pathogenesis by promoting cancer stem cell (CSC) proliferation, postponing apoptosis, continuing the cell cycle, and promoting invasion, angiogenesis, and metastasis. Similarly, miRNAs influence important CC-related molecular pathways, such as the PI3K/AKT/mTOR signaling pathway, Wnt/β-catenin system, JAK/STAT signaling pathway, and MAPK signaling pathway. Moreover, miRNAs affect the response of CC patients to chemotherapy and radiotherapy. Consequently, this review aims to provide an acquainted summary of onco miRNAs and tumor suppressor (TS) miRNAs and their potential role in CC pathogenesis and therapy responses by focusing on the molecular pathways that drive them.

## 1 Introduction

CC is one of the most prevalent gynecological carcinomas, posing a significant threat to the female reproductive system and is the primary cause of cancer-related mortality worldwide (Holcakova et al., 2021). According to epidemiological research on CC, there are an estimated 569,847 new cases and 311,365 deaths worldwide, annually. Alarmingly, projections indicate that by 2030, the incidence of CC is expected to increase by approximately 50% (Kakotkin et al., 2023), underscoring the urgency of understanding its underlying causes. Human papillomaviruses (HPV) is a DNA virus known to infect epithelial cells, and it's the most common sexually transmitted infection globally. While many HPV infections resolve on their own without causing any symptoms or long-term effects, persistent infection with high-risk HPV types, such as HPV-16 and HPV-18, can lead to the development of CC (Liu et al., 2019). The relationship between HPV and cervical cancer is multifaceted. HPV contributes to cancer progression through various mechanisms, including the modulation of cellular processes and the interference with key regulatory proteins within the host cell (Oyervides-Muñoz et al., 2018). The oncoproteins E5, E6, and E7 encoded by HPV play critical roles in this process. Oncoprotein E5 modulates cellular signaling pathways like EGFR, promoting increased cell proliferation and survival, thus aiding in maintaining the transformed phenotype of HPV-infected cells (Pal and Kundu, 2020). E6 facilitates the degradation of the tumor suppressor protein p53, inhibiting apoptosis and enabling HPV-infected cells to evade cell cycle control, thereby promoting proliferation. E7 interacts with and promotes the degradation of the retinoblastoma (Rb) tumor suppressor protein, disrupting normal cell cycle regulation and leading to uncontrolled cell division and tumor formation (Taghizadeh et al., 2019). Additionally, Epigenetic alterations induced by HPV oncoproteins contribute significantly to the oncogenic process. HPV oncoproteins, such as E6 and E7, can disrupt normal epigenetic regulation in several ways. For instance, E6 and E7 can directly interact with cellular proteins involved in epigenetic regulation, altering their function and leading to aberrant DNA methylation patterns or histone modifications (Sen et al., 2018). Additionally, these oncoproteins can indirectly affect epigenetic processes by promoting the expression of specific non-coding RNAs, such as microRNAs or long non-coding RNAs, which can further modulate gene expression patterns. This disruption of epigenetic regulation by HPV oncoproteins can lead to the silencing of tumor suppressor genes or the activation of oncogenes, ultimately driving the development and progression of HPV-associated cancers like cervical cancer (Ferreira and Esteller, 2018). Although CC is commonly linked with HPV infection, not all diagnosed cases test positive for HPV (Saslow et al., 2012), suggesting the involvement of additional factors in CC development. Despite notable progress in surgical, chemotherapy, and radiation treatments, CC remains complex. Recent studies have highlighted specific dysregulated microRNAs (miRNAs) in CC, suggesting their potential as diagnostic and prognostic biomarkers (He et al., 2016). These findings hold promise for advancing more targeted and personalized strategies to address CC.

**FIGURE 1:**
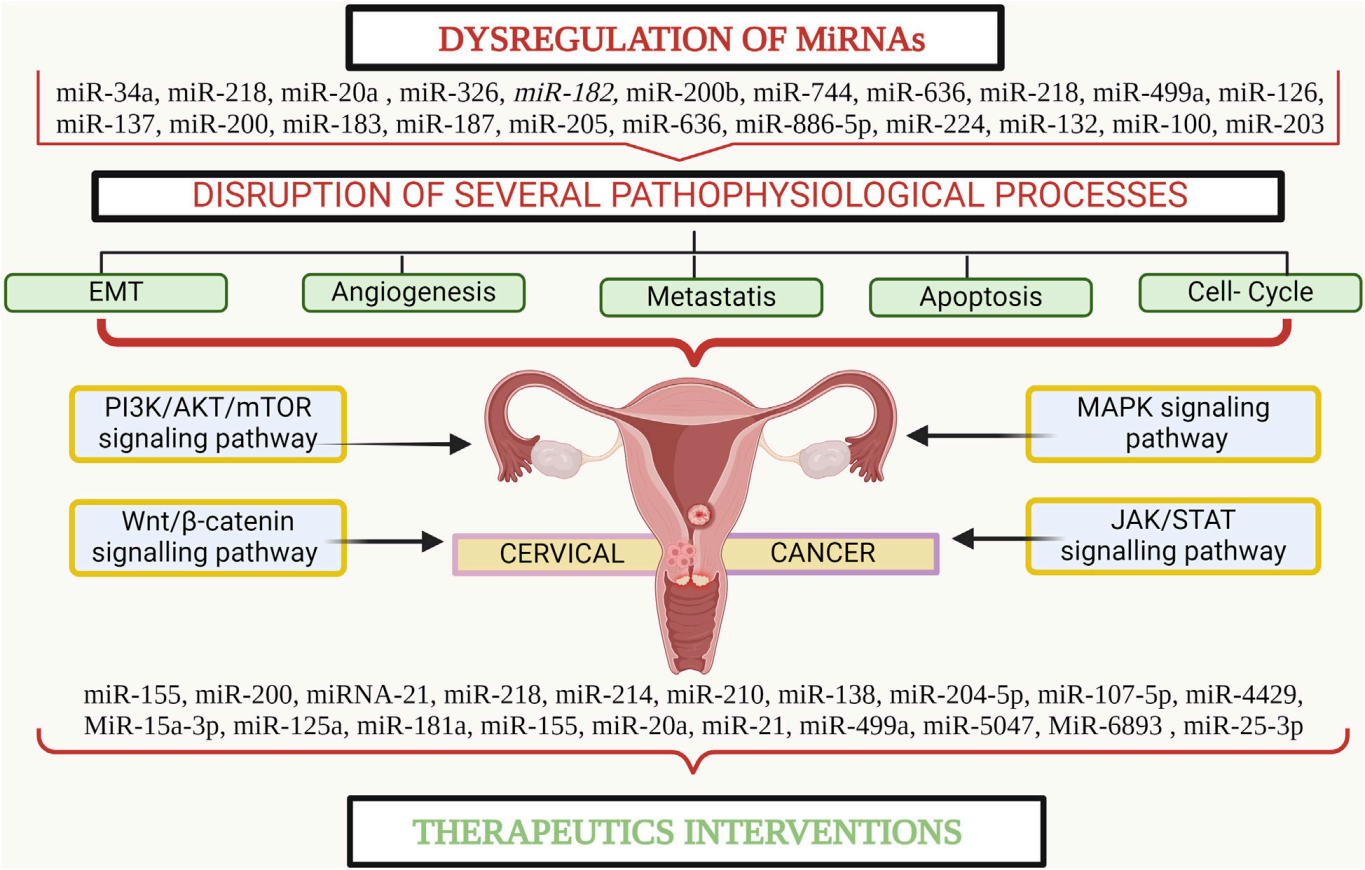
miRNA plays a crucial role in the pathogenesis and progression of cervical cancer. Created with BioRender.com.

miRNAs, small non-coding RNAs, regulate gene expression and are associated with tumorigenesis and other biological processes. The first description of the role of miRNAs in cancer was published in 2002 (Acunzo et al., 2015). Studies have indicated that particular miRNAs are dysregulated in CC, implying that they may serve as biomarkers for diagnosis, prognosis, and therapeutic targets. Recent research has uncovered a multitude of miRNAs that play critical roles in CC pathogenesis and therapeutic response (Abbas et al., 2021). These miRNAs have emerged as potent regulators of gene expression, influencing key cellular processes, such as cell proliferation, apoptosis, angiogenesis, and metastasis (Sharma et al., 2014). Additionally, studies have revealed their crucial involvement in the dysregulation of key signaling pathways involved in the evolution of CC, including the PI3K/AKT/mTOR, Wnt/β-catenin, MAPK, and JAK/STAT pathways (Hemmat et al., 2020). Their complex effects highlight their potential as therapeutic targets in the fight against CC. miRNAs, with their compact size (∼22 nucleotides), possess unique qualities that make them readily isolable and easily identifiable in various bodily samples, including tissues, blood, and bodily fluids, and are relatively resistant to degradation (Chevillet et al., 2014). Moreover, their intricacies are intertwined with those of cancer stem cells (CSCs), a subset of cancer cells endowed with distinctive self-renewal and differentiation capabilities. Within CSCs, specific miRNAs act as catalysts, fuelling self-renewal, migration, and resistance to therapy, thus garnering attention for their pivotal roles in both tumor initiation and progression (Khan et al., 2019). Furthermore, the discovery of miRNAs associated with therapeutic response in CC offers new avenues for personalized treatment strategies. By understanding how these miRNAs influence the sensitivity or resistance to different therapeutic approaches, clinicians can tailor treatment regimens to individual patients, potentially improving overall outcomes (Figure 1) (Gandellini et al., 2011; Gong et al., 2014). Therefore, this comprehensive review aims to shed light on the intricate role of miRNAs in the biogenesis of CC and elucidate their impact on dysregulated signaling pathways critical to CC development. Furthermore, it underscores the pivotal roles played by specific oncogenic and suppressor miRNAs in either hindering or promoting therapeutic interventions for CC, positioning miRNAs as promising diagnostic and prognostic markers, and warranting further investigation. By consolidating these insights, this study not only advances our understanding of the molecular complexities underlying CC progression but also lays the foundation for more targeted and precise management strategies. The implications of this review are poised to significantly shape future CC research endeavours, driving the formulation of more efficacious approaches for its treatment and care.

## 2 Canonical and non-canonical pathway of miRNAs biogenesis

**FIGURE 2:**
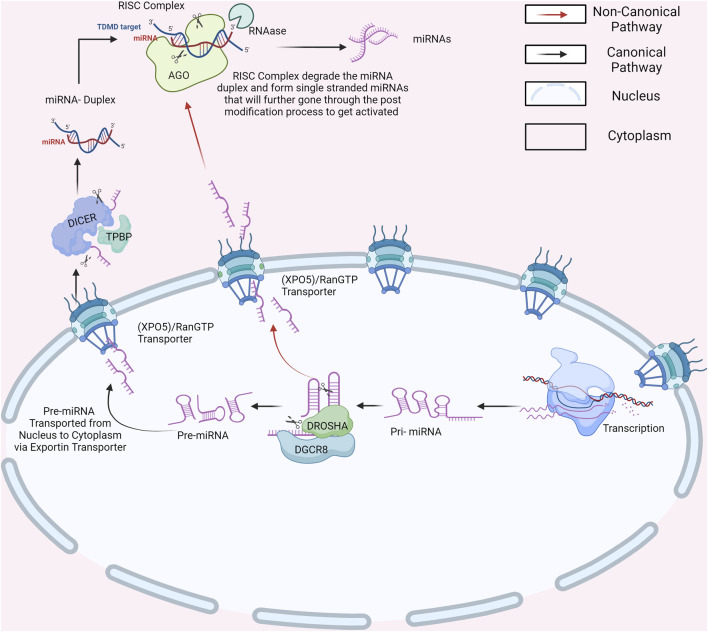
Canonical and Non-Canonical Pathways of miRNA Biogenesis. Created with BioRender.com.

miRNA biogenesis is a complicated set of events that result in the generation of mature miRNAs capable of influencing gene expression. miRNAs are involved in many biological processes and are essential for balanced human growth. Abnormalities in miRNA expression have been linked to a variety of diseases, emphasizing their importance in disease pathophysiology (Peng and Croce, 2016). Furthermore, miRNAs are not restricted to cells but are also released into extracellular fluids. This extracellular release of miRNAs adds another degree of complexity to their regulatory actions while also emphasizing their potential as diagnostic and prognostic indicators for diverse illnesses (Ortiz-Quintero, 2020). Extracellular miRNAs act as signaling molecules, facilitating cell-to-cell communication and serving as promising biomarkers for various illnesses, notably cancer (Conti et al., 2020). Hence, understanding the intricacies of miRNA biogenesis is crucial to effectively managing the onset and progression of severe diseases. miRNA biogenesis begins with the processing of RNA polymerase II/III transcripts, either post-transcriptionally or co-transcriptionally, which initiates the production of miRNAs (Panda and Prajapati, 2024). Around half of the identified miRNAs arise from within genes, mainly from introns, with a smaller portion originating from exons. The other half, however, are intergenic, meaning they are transcribed independently, regulated by their own promoters (Bofill-De Ros and Vang Ørom, 2024). miRNAs are typically grouped as families unless they are transcribed into clusters. These clusters were defined as single-long transcripts that might share seed regions. The biogenesis of miRNAs can be divided into two categories: canonical and non-canonical pathways (Figure 2) (Abdelfattah et al., 2014). The canonical biogenesis pathway is the primary route for miRNA processing. In this pathway, primary miRNAs (pri-miRNAs) are transcribed from their respective genes and subsequently transformed into precursor miRNAs (pre-miRNAs) by the action of the microprocessor complex (MacFarlane and R Murphy, 2010). This complex comprises the RNA binding protein DiGeorge Syndrome Critical Region 8 (DGCR8) and the ribonuclease III enzyme Drosha. DGCR8 identifies specific motifs, including an N6-methyladenylated GGAC, within pri-miRNA (Rani and Sengar, 2022). Drosha subsequently cleaves the pri-miRNA duplex at the base of its characteristic hairpin structure, leading to the formation of a 2-nucleotide 3 overhang on the pre-miRNA. Following their generation, pre-miRNAs undergo export to the cytoplasm, facilitated by the exportin 5 (XPO5)/RanGTP complex (Shirley et al., 2022). In the cytoplasm, to process pre-miRNAs, Dicer binds with a double-stranded RNA-binding domain (dsRBD) protein known as TAR RNA-binding protein (TRBP), eliminating the terminal loop and forming miRNA duplexes (Ergin and Çetinkaya, 2022). The miRNA duplex, produced by Dicer, is loaded onto an Argonaute (AGO) protein, resulting in an effector complex known as the RNA-induced silencing complex (RISC). TRBP and AGO proteins go through post-translational changes, which affect their capacity to control Dicer processing, RISC formation, and miRNA activity. The guide strand of the miRNA-RISC complex directs RISC to target mRNAs by forming complementary base pairs, resulting in mRNA destabilization, translational repression, or cleavage (MacFarlane and R Murphy, 2010). This process, mediated by AGO proteins, is central to the functionality of RISC, enabling it to recognize and interact with target mRNAs, thus initiating post-transcriptional gene silencing, which plays a pivotal role in directing target mRNA recognition and gene regulation. Precise function of the miRNA-RISC complex in fine-tuning gene expression (Iwakawa and Tomari, 2022). This intricate process ultimately regulates gene expression and precisely orchestrates diverse cellular functions. Notably, the selection between 5p and 3p strands of the mature miRNA duplex for loading into AGO is a dynamic process influenced by factors such as thermodynamic stability and nucleotide composition (Zaporozhchenko et al., 2020). The guide strand, chosen for its ability to recognize target miRNAs and regulate genes, earns its name. Meanwhile, its counterpart, the passenger strand, is discarded. This meticulous selection showcases the miRNA machinery’s adaptability to the cellular environment (Treiber et al., 2019). Alternatively, researchers have uncovered an additional noncanonical pathway for miRNA biogenesis. Some miRNAs bypass Drosha-mediated processing by undergoing direct cleavage by Dicer or by splicing-independent processing of introns (Yang and Lai, 2011). In certain scenarios, miRNAs emerge independently of Dicer with the help of endonucleases or exonucleases. Mirtrons, for instance, are intriguing miRNAs generated from spliced introns that form hairpin structures akin to pre-miRNAs, effectively circumventing Drosha processing (Cipolla, 2014). Additionally, small nucleolar RNAs (snoRNAs) and transfer RNAs (tRNAs) emerge as unlikely contributors to non-canonical miRNA biogenesis, unveiling the diverse origins of these regulatory molecules. Moreover, certain pre-miRNAs produce pre-miRNAs with suboptimal structures, necessitating monouridylation by TUTase for efficient processing (Havens et al., 2012). In this intricate pathway, a truncated pre-miRNA is generated by Drosha, shuttled to the cytoplasm, and loaded onto Argonaute 2 (AGO2) without Dicer involvement. Subsequent cleavage by AGO2 and trimming by the exonuclease PARN further refined the miRNA, highlighting the multifaceted nature of miRNA biogenesis (Abdelfattah et al., 2014; Santovito and Weber, 2022). These diverse pathways underscore the complexity and adaptability of cellular mechanisms for generating functional miRNAs for precise post-transcriptional gene regulation. As miRNAs undergo biogenesis, they have the potential to influence a variety of cellular processes including cell proliferation, differentiation, and apoptosis (Herrera-Carrillo and Berkhout, 2017). Altered miRNA function can disturb gene regulation, fueling abnormal cell behavior and advancing various cancers, including CC (Iorio and Croce, 2012). In cancer cells, miRNAs may act as oncogenes, promoting tumor growth and metastasis, or as TS, inhibiting tumorigenesis and metastasis. Dysregulated miRNA expression can affect the functionality of genes involved in essential cancer-related processes such as cell cycle control, apoptosis, DNA repair, and epithelial-mesenchymal transition (EMT) (Medina and Slack, 2008). Therefore, the complex connection between miRNA biogenesis and cancer pathogenesis emphasizes the critical role of miRNAs in driving oncogenic processes as well as their possible use as diagnostic and therapeutic targets in the management of cancer.

## 3 Role of miRNAs in initiation, proliferation, and progression of cervical cancer

miRNAs have emerged as important participants in the development, proliferation, and progression of CC. These short non-coding RNA molecules have been shown to control gene expression at the post-transcriptional level, impacting a variety of cellular processes implicated in cancer formation. Numerous studies have discovered distinct miRNAs that are dysregulated in CC, resulting in the disruption of essential signaling networks (Ortiz et al., 2023). Furthermore, miRNAs have been shown to influence the expression of genes involved in cell cycle regulation, apoptosis, angiogenesis, and metastasis, all of which play key roles in tumor initiation, proliferation, and progression (Doghish et al., 2023). Moreover, miRNAs have been implicated in the regulation of EMT, a process that is crucial for metastasis and cancer progression. Certain miRNAs act as either suppressors or promoters of EMT-associated pathways, thereby affecting the invasive potential of CC cells. Overall, miRNAs play a significant role in the initiation, proliferation, and progression of CC by regulating gene expression and influencing key cellular processes involved (Jafri et al., 2017). Moreover, dysregulation of specific miRNAs in CC can lead to the activation of critical signaling pathways, contributing to tumor progression. Several signaling pathways such as the PI3K/AKT/mTOR pathway, the Wnt/β-catenin pathway, the MAPK/ERK pathway, and JAT/STAT pathways are regulated by miRNAs in CC, further highlighting the importance of miRNAs in cancer development (Hasan et al., 2023).

Furthermore, alterations in miRNA expression levels disrupt the balance between oncogenic and tumor-suppressor proteins, thereby influencing cellular development and promoting tumorigenesis (Sharma et al., 2014). The intricate interplay between these factors contributes to the initiation and progression of cancer by dysregulating critical cellular processes such as proliferation, deletion, and mutation of miRNA loci (Jafri et al., 2017). This dysregulation is often attributed to perturbations in the transcription factors and epigenetic silencing mechanisms. Moreover, a distinct subset of miRNAs known as epi-miRNAs exert regulatory control over tumor suppressor genes indirectly by manipulating epigenetic machinery effectors, including DNA methyltransferases, histone deacetylases, and genes within the polyoma suppressor complex (Chengizkhan et al., 2024). This complex regulatory landscape underscores the multifaceted role of miRNAs in cancer development and highlights their potential as targets for therapeutic interventions (Sharma et al., 2014). Additionally, the mechanisms driving CC have unveiled a deeper understanding of the role of miRNAs. Interestingly, a study examines pri-miR-34a levels in various cervical tissues, revealing significant reduction in cervical intraepithelial neoplasia (CIN) and CC compared to normal epithelium, even in early-stage lesions. Additionally, HR-HPV infection correlates with lower pri-miR-34a expression. Experimental findings suggest that HR-HPV E6 induces pri-miR-34a downregulation, potentially via a p53-dependent pathway, observed in transfected cells. These results underscore pri-miR-34a’s role as an early indicator of CC development, implicating HR-HPV E6 in its pathogenesis (Li et al., 2010). Moreover, another study demonstrated that miR-20a is significantly upregulated in CC patients, correlating with lymph node metastasis, histological grade, and tumor size. Inhibiting miR-20a with stable anti-miR-20a cell lines suppresses tumor progression by affecting cell cycle, apoptosis, and metastasis *in vitro* and *in vivo*. Additionally, miR-20a directly targets TIMP2 and ATG7, suggesting its role in regulating CC proliferation, migration, and invasion. These findings propose miRNAs as potential therapeutic agents for CC (Bao et al., 2013). Furthermore, a study investigated the role of miR-499a in CC development, finding its significant upregulation in CC cells. Overexpression of miR-499a promotes cancer cell proliferation, migration, invasion, and resistance to apoptosis, while inhibition suppresses these effects. The study identifies SOX6 as a direct target of miR-499a, mediating its oncogenic effects. Inhibiting miR-499a enhances the anticancer effects of cisplatin in a mouse model. These findings propose miR-499a as a potential therapeutic target in cervical cancer (Chen Y. et al., 2020). Moreover, a study investigates microRNA-137 (miR-137) in CC, assessing its expression, clinical relevance, and functional role. MiR-137 downregulation is found in CC cells and tumors, correlating with shorter overall survival in patients. Upregulation of miR-137 inhibits CC proliferation and migration *in vitro* and *in vivo*, potentially by targeting enhancer of zeste homolog 2 (EZH2). EZH2 overexpression reverses miR-137-induced tumor suppression, suggesting its involvement in miR-137-mediated CC regulation. This highlights miR-137 as a negative biomarker for CC prognosis and a potential therapeutic target (Zhang et al., 2018). The study investigates the role of miR-187, a newly identified cancer-related microRNA, in CC. It reveals decreased miR-187 levels in CC tissues and cell lines, with low miR-187 associated with decreased overall survival and progression-free survival rates in patients. Overexpression of miR-187 inhibits proliferation and promotes apoptosis in CC cells, while knockdown enhances proliferation and inhibits apoptosis. Forced expression of miR-187 suppresses subcutaneous tumor growth in mice. Additionally, FGF9 is identified as a downstream target of miR-187, and targeting FGF9 is crucial for miR-187s tumor-suppressive effects in CC cells (Liang et al., 2023). In addition to this (Table 1), highlights several miRNAs that play pivotal roles in the initiation, proliferation, and progression of CC, emphasizing their critical impact on key aspects of CC progression.

**TABLE 1:** miRNAs affects the progression of cervical cancer.

miRNA	*In vitro*/ In silico/*In vivo*	Mechanism of action	Target genes	Oncomirna/ suppressor	Reference
miR-34a	*In vitro*	• P53-dependent pathway • Cell cycle progression • Cellular senescence	P18Ink4c, CDK4, CDK6, Cyclin A, E2, E2F1, BCL2, BIRC3	Oncomirna	Li et al. (2010)
miR-218	*In vitro*	• Focal adhesion	LAMB3	suppressor	Yamamoto et al. (2013)
miR-20a	*In vivo* and *In vitro*	• lymph node metastasis	ATG7 and TIMP2	Oncomirna	Bao et al. (2013)
miR-326	*In vivo* and *In vitro*	• Cell proliferation	TCF4	suppressor	Zhang et al. (2020)
miR-182	*In vivo* and *In vitro*	• Disrupting cell proliferation • Apoptosis • Cell cycle pathways	FOXO1	Oncomirna	Tang et al. (2013)
miR-200b	*In vitro*	• Induced the decrease of cell ability • Increased cell apoptosis • Attenuated ability of cell migration and invasion	FoxG1	Oncomirna	Choi et al. (2022)
miR-744	*In vitro*	• Apoptosis induction	Bcl-2,	suppressor	Chen and Liu (2016)
miR-636	*In vitro*	• Represses cell survival	CDK6/Bcl-2	suppressor	Hu et al. (2020)
miR-218	*In vitro*	• Reduced the proliferation	cisplatin (CDDP)	suppressor	Li et al. (2012)
miR-499a	*In vivo*	• Enhanced the proliferation • Cell cycle progression • Colony formation • Apoptosis resistance • Migration and invasion	SOX6	Oncomirna	Chen et al. (2020a)
miR-137	*In vitro* and *In vivo*	• Cell proliferation and migration	EZH2	Oncomirna	Zhang et al. (2018)
miR-133b	*In vitro*	• Enhances cell proliferation • Colony formation	MST2, CDC42, ERK1 and ERK2, RHOA, AKT1	Oncomirna	Qin et al. (2012)
miR-200	*In vitro*	• Metastatic inhibit (EMT)	ZEB1, ZEB2, Sip 1	suppressor	Gregory et al. (2008)
miR-372	*In vitro*	• Cell growth induce arrest in S/G2 phase of cycle	CDK1, Cyclin A1	Oncomirna	Tian et al. (2011)
miR-187	*In vitro*	• Inhibition of the growth of CC cells by targeting FGF9	FGF9	suppressor	Liang et al. (2023)
miR-205	*In vitro*	• Promotion of angiogenesis • Tumour progression by activating the Akt signalling pathway via TSLC1 upregulation	TSLC1	suppressor	Zhang et al. (2019a)
miR-636	*In vitro*	• Inhibition of cell proliferation • induction of cell apoptosis by targeting CDK6 and Bcl-2	CDK6 and Bcl-2	suppressor	Hu et al. (2020)
miR-145	*In vitro*	• Cell motility	IGF-1	suppressor	Wang et al. (2015)
miR-886-5p	*In vitro*	• Cell transformation and B progression	AX	Oncomirna	Li et al. (2011a)
miR-873-5p	In silico and *In vitro*	• Suppressed the expressions of Jag1, Maml2 and Hey1	ZEB1	Oncomirna	Wen et al. (2021)
miR-224	*In vitro*	• Associated with aggressive progression and poor prognosis	RASSF8	Oncomirna	Huang et al. (2016)
miR-126	*In vitro*	• Promotion of CC advancement through PI3K/AKT/mTOR pathway activation	ZEB1	suppressor	Xu et al. (2019)
miR-214	*In vitro*	• Promotion of CC advancement through PI3K/AKT/mTOR pathway activation	Bcl2l2	suppressor	Wang et al. (2013)
miR-100	*In vitro*	• Growth, cell cycle and apoptosis	PLK1	suppressor	Li et al. (2011b)

### 3.1 Role of miRNAs in cervical cancer progression

miRNAs contribute to dynamic and complex processes involved in CC progression. It may cover various aspects, including modulation of signalling pathways and regulation of key cellular processes. The interplay between miRNAs and specific molecular targets suggests the potential role of miRNAs in CC progression (Melo and Esteller, 2011). Approximately half of all miRNAs are located within genomic regions implicated in cancer, underscoring their dual roles as oncogenes, which promote cancer development, and TS genes, which inhibit tumor growth. This distinct nature underlines the complex regulatory processes of miRNAs in CC progression (Xu et al., 2016). Dysregulation of specific miRNAs has been associated with various aspects of CC progression, including cell proliferation, invasion, and metastasis (Pardini et al., 2018). The expression profiles of miRNAs vary significantly depending on the histological type of the tissue and pathological or non-pathological conditions. This discrepancy is evident in the distinct expression patterns observed in normal and cancerous tissues (Causin et al., 2021). Therefore, understanding the expression patterns of several miRNAs in the context of CC is crucial for understanding their implications in cancer progression.

Interestingly, a study retrieved data from 24 studies to determine the role of miRNAs in CC and found that the upregulation and downregulation of miR-29a and miR-21 were significantly linked with the progression of CC (Xu et al., 2016). Similarly, another study has been conducted and revealed that miR-132 is a pivotal regulator in the progression of CC, exerting a significant influence on tumor growth and advancement. It plays a crucial role in suppressing the expression of RDX oncogene, thereby facilitating the proliferation and progression of CC cells. This mechanism underscores the intricate involvement of miR-132 in driving the aggressive nature of CC and highlights its potential as a therapeutic target for intervention strategies aimed at impeding disease progression (Pardini et al., 2018). Furthermore, to investigate the clinical importance of miR-224, a study performed an experiment using 126 pairs of CC cell lines along with normal cells and observed upregulation of miR-224, which was aggressively associated with the progression of CC (Shen et al., 2013). Additionally, a study investigated the clinical relevance of miR-145 in CC by analysing 114 pairs of human CC tissue samples and adjacent normal tissues. Using reverse transcription-quantitative polymerase chain reaction (RT-qPCR) assays, observed a significant downregulation of miR-145 expression in CC cell lines. This suggests a potential association between decreased miR-145 expression and the progression of CC (Wang et al., 2015). RT-qPCR was used to assess the expression levels of miRNA-873-5p in CC specimens and cell lines. These findings provided compelling evidence indicating that decreased expression of miRNA-873-5p serves as an unfavorable prognostic factor for CC patients (Wen et al., 2021). To uncover a unique miRNA fingerprint for CC and cervical intraepithelial neoplasia (CIN), a study employed a cutting-edge miRNA microarray was used to delve into the intricate world of miRNA expression profiles, comparing CC, CIN, and normal cervical tissues, and Real-time RT-PCR was used to validate the expression of miRNAs. Interestingly, functional studies were performed to determine the downregulation of miR-218 and upregulation of miR-21 among all miRNAs and concluded that these miRNAs may be involved in the progression of cervical neoplasm (Zeng et al., 2015). Furthermore, to delve deeper into the functional significance of miR-200b in CC progression, a study was conducted to elucidate its involvement in CC development. This study analyzed 30 paired CC samples to explore the role of miR-200b in CC. Remarkably, these findings revealed a notable upregulation of miR-200b in cancer tissues, accompanied by the downregulation of FoxG1, which was subsequently identified as a target gene of miR-200b (Zeng et al., 2016). Moreover, to unveil the pathogenic mechanisms underlying CC, an intriguing investigation revealed the upregulation of miR-182 and miR-183 in CC cell lines. Conversely, the expression of nine mRNAs (miR-211, 145, 223, 150, 142-5p, 36, 328, 195, 199b, and 142-3p) was consistently identified across CC cell lines. Particularly, heightened expression of miRNA-182 was also observed in primary CC, correlating with the progression of advanced CC. These findings implicate miR-182 as an onco-miRNA in CC progression, and its dysfunction is linked to CC pathogenesis by disrupting cell proliferation (Tang et al., 2013).

### 3.2 miRNAs affect the cell cycle during cervical cancer progression

The intricate interplay between miRNAs and the cell cycle stands as a pivotal determinant in the progression of CC. Therefore, understanding the consequences of miRNAs on the complicated procedure of cell cycle regulation is crucial as errors in this pathway play an integral part in the emergence and progression of CC (Choi et al., 2022). However, emerging research has shed light on the multifaceted roles of miRNAs in modulating the cell cycle dynamics in CC. Through their regulatory effects on the cell cycle, miRNAs wield significant influence over the progression and proliferation of CC cells (Shen et al., 2020).

In this context, examining the intricate interaction between miRNAs and the cell cycle reveals unique insights into the pathophysiology of CC. As a result, researchers have delved into the complex web of CC development to determine the functional significance of miRNAs in interrupting the cell cycle. Amidst this exploration, Baicalein, known for its anti-cancer properties in CC treatment, was investigated for its mechanism of action involving circular RNA (circRNA) hippocampus abundant transcript 1 (circHIAT1) and miRNA-19a-3p (miR-19a-3p) (Hu et al., 2021). Cell viability and colony formation assays revealed baicalein’s inhibition of CC cell growth and cell cycle progression, coupled with enhanced apoptosis. Notably, miR-19a-3p downregulation in baicalein-treated CC cells and its overexpression mitigated baicalein-induced inhibition of CC progression (Hu et al., 2021). Furthermore, a study focused on unravelling the role and molecular mechanism of miRNA-29a in CC progression. Through a series of experiments utilizing various molecular and cellular techniques, the researchers found that miR-29a expression was diminished in CC tissues and cells, correlating negatively with hypermethylation of the p16 promoter. Additionally, functional assays revealed that miR-29a inhibited cell proliferation and induced cell cycle arrest in CC cells. Mechanistically, miR-29a was found to modulate the methylation pattern of the p16 gene by targeting DNA methyltransferases (DNMT)3A and (DNMT)3B (Robaina et al., 2015). These findings shed light on the epigenetic regulation of the tumour suppressor p16 by miR-29a, presenting a novel mechanism in CC progression (Robaina et al., 2015). A study, through a comprehensive analysis of the cancer genome atlas (TCGA) data, unveiled a significant reduction in miR-140-3p levels in CC tissues. Subsequently, RT-qPCR assays confirmed this negative correlation between miR-140-3p expression and both CC tissues with various cell lines. To elucidate the specific role of miR-140-3p, researchers employed miRNA mimics to enforce its expression in Caski and C33A cells. Remarkably, the overexpression of miR-140-3p notably hindered CC cell proliferation, as evidenced by Cell Counting Kit (CCK-8) assays. Further validation through Western blot analysis revealed a consequential induction of cell cycle arrest, supported by decreased levels of cell cycle-related proteins Cyclin A, Cyclin B1, and Cyclin D1 (Ma et al., 2020). Moreover, the downregulation of miR-372 was observed in cervical carcinoma tissues adjacent to normal cervical tissues. An investigation using growth curve and Fluorescence-Activated Cell Sorting (FACS) tests showed that aberrant expression of miR-372 causes arrest in the S/G2 stages of the cell cycle and decreases cell growth in HeLa cells. The study used bioinformatics predictions to identify cyclin-dependent kinase 2 (CDK2) and cyclin A1 as putative targets of miR-372. A fluorescence reporter test was used to confirm this hypothesis (Tian et al., 2011).

### 3.3 miRNAs affect apoptosis during cervical cancer progression

The influence of miRNAs on apoptosis appears to be a critical factor in the intricate domain of CC development and requires further exploration (Wang and Chen, 2019). Interestingly, several studies have demonstrated the crucial role of miRNAs in modulating apoptosis in CC development. Apoptosis is a basic mechanism that maintains homeostasis and eliminates aberrant cells, including cancer cells (Miao et al., 2020). Therefore, dysregulation of this fundamental mechanism is a hallmark of the progression of numerous carcinomas such as CC. Hence, outlining the intricate relationships between miRNAs and apoptosis is essential for understanding the molecular processes that drive the advancement of CC. Meanwhile, a study investigated the oncogenic role of miRNA in nine CC cell lines and observed that elevated expression of miR-181a significantly modulated apoptosis in cell lines. Later, the inhibited expression of miR-181a promoted apoptosis in CC cells, suggesting that miR-181a might be an oncogene in CC cells (Xu et al., 2016). The mRNA and protein expression levels of Bcl-2 in HeLa cells were increased by miR-34a-5p suppression, but decreased by miR-34a-5p overexpression. Bcl-2 is a direct target gene of miR-34a-5p and participates in the effects of miR-34a-5p on HeLa cell viability, migration, invasion, and apoptosis. Suppression of miR-34a-5p promoted the viability, migration, and invasion of HeLa cells by increasing the expression of Bcl-2 (Wang X. et al., 2018). Moreover, overexpression of Bcl-2 significantly promoted cell viability, migration, and invasion and had no influence on cell apoptosis. The suppression of Bcl-2 showed the opposite effect, with an increase in apoptosis. Therefore, Bcl-2 expression is downregulated when miR-34a-5p is overexpressed, which prevents human CC cells from proliferating and promotes their death (Wang X. et al., 2018).

Similarly, another study demonstrated the intricate role of miR-7 in HeLa and C-33A cell lines, shedding light on its profound impact on cell viability and apoptosis. The results revealed a remarkable association. Overexpression of miR-7 significantly suppressed cell viability coupled with a marked increase in apoptosis. In contrast, miR-7 inhibition had the opposite effects, emphasizing its role in cellular dynamics. Additionally, an X-linked inhibitor of apoptosis protein (XIAP), an oncogene, was shown to be a novel target of miR-7 in HeLa and C-33A cells, its effects of miR-7 were mitigated by the ectopic production of XIAP. These findings suggest that miR-7 regulates apoptosis and malignancy by targeting XIAP, thereby offering therapeutic avenues for cancer treatment (Liu et al., 2013). Furthermore, miR-148a plays a crucial role in regulating the growth, apoptosis, invasion, and migration of CC cells by targeting the regulator of ribosome synthesis 1 (RRS1). The expression levels of miR-148a and RRS1 were analyzed in CC tissues and cell lines. Downregulation of miR-148a and upregulation of RRS1 have been observed in CC tissues and cells, correlating with poor clinicopathological characteristics. Functional assays revealed that upregulation of miR-148a inhibited cell proliferation, migration, and invasion while promoting apoptosis in CC cells. Furthermore, RRS1 was identified as a direct target of miR-148a, and miR-148a negatively regulated RRS1 expression. These findings underscore the tumor-suppressive function of miR-148a in CC development by modulating RRS1 expression, suggesting its potential as a therapeutic target for CC (Zhang Y. et al., 2019). miR-150 exerts a significant influence on CC cell survival and growth, and its inhibition leads to the suppression of these actions. Furthermore, miR-150 drives cell cycle progression from G1/G0 to S phase, thereby enhancing cell growth. Notably, miR-150 modulated the expression of key cell cycle- and apoptosis-related genes, including CyclinD1, p27, BIM, and FASL. Additionally, miR-150 directly targets the 3′-UTR of FOXO4, a regulator of CyclinD1, p27, BIM, and FASL expression, thereby reducing FOXO4 expression levels. This multifaceted regulatory role underscores the significance of miR-150 in CC progression (Li et al., 2015).

Moreover, *in vitro* investigations showed that a decreased level of miR-200b lowered cell ability and cell apoptosis and altered the cell migration ability in both C33A and HeLa cells (Choi et al., 2022). Additionally, to distinguish the role of miR-636 in CC progression or inhibition, a study has shown that miR-636 is significantly downregulated in CC cells, and *in vitro* results suggested that the upregulation of miR-636 can inhibit cell proliferation and induce cell apoptosis. Moreover, cyclin-dependent kinase 6 (CDK6) and B-cell lymphoma 2 (Bcl-2) are targets of miR-636 (Hu et al., 2020). Bioinformatic analysis of miR-122 identified RAD21 as its target gene. A study revealed that overexpression of miR-122 induces cell cycle arrest and promotes apoptosis by targeting RAD21 (Yang et al., 2022). Regulation of apoptosis showed that miR-146a exerts an effect on the regulation of Th17 cell differentiation, and further studies have revealed that miR-146a enables its target gene TRAF6 to regulate CC cell growth and apoptosis through the NF-kB signalling pathway (Li T. et al., 2019).

### 3.4 miRNAs affect migration, EMT, invasion, and metastasis during cervical cancer progression

Numerous investigations have unveiled the critical involvement of miRNAs in CC invasion and metastasis through their regulation of key pathways, notably the Notch, Wnt/β-catenin, and phosphoinositide-3 kinase (PI3K)-Akt pathways (Wang et al., 2016). Additionally, miRNAs play a crucial role in modulating EMT, further contributing to the metastatic cascade in CC. Several findings offer novel perspectives on the complex role of miRNAs in driving the pathogenesis of metastatic CC, underscoring their potential as therapeutic targets and prognostic markers in combating this disease (Wang and Chen, 2019). As a result, a study experimented to determine the relationship between miR-20a and progression of CC and analysed the upregulated expression of miR-20a in CC cells in comparison to normal cells by *in vitro* and *in vivo* analysis (Zhao et al., 2015). Furthermore, inhibition of miR-20a effectively halted tumour progression by influencing cell cycle regulation, apoptosis, and metastasis both *in vitro* and *in vivo*. Additionally, TIMP2 and ATG7 were identified as direct targets of miR-20a through luciferase assays and western blot analysis. These findings underscore the role of miR-20a in cervical tumorigenesis, particularly in lymph node metastasis (Zhao et al., 2015). Moreover, an investigation experimented with elucidating the correlation of miR-218 with CC and discovered the downregulation of miR-218 especially in metastatic cancer tissues. Furthermore, miR-218 expression was discovered to be related to the clinicopathological features of patients with CC. *In vitro*, overexpression of miR-218 inhibited the motility, invasion, and EMT of CC cells. Moreover, miR-218 directly suppressed the expression of SFMBT1 and DCUN1D1 mRNAs by targeting their 3′UTRs. While DCUN1D1 overexpression boosted migration and invasion without producing EMT, elevated levels of SFMBT1 produced EMT and increased migration and invasion (Li et al., 2012). Moreover, miR-204-5p displays reduced expression levels in CC cells, leading to diminished cellular functionalities such as proliferation, invasion, migration, and EMT. Through functional assays, it was established that the upregulation of miR-204-5p exerts a suppressive effect on these cancerous processes. Notably, transcription factor AP-2 alpha (TFAP2A) emerged as the primary target gene affected by miR-204-5p, with TFAP2A found to transcriptionally repress miR-204-5p in CC cells. Molecular mechanism assays confirmed the reciprocal regulation between TFAP2A and miR-204-5p. Subsequent rescued-function assays revealed that overexpression of TFAP2A could reverse the inhibitory effects of miR-204-5p upregulation on cellular processes. Altogether, the miR-204-5p/TFAP2A feedback loop enhances the proliferative and motility capabilities of CC cells, underscoring a novel regulatory mechanism with potential implications for CC therapy (Bao et al., 2013). In CC patients, reduced expression of miR-125a is observed, correlating inversely with tumour size, FIGO stage, and preoperative metastasis. Kaplan-Meier analysis indicates that higher miR-125a expression predicts better outcomes for patients. Through dual luciferase assays, the STAT3 gene is identified as a direct target of miR-125a. Functional investigations demonstrate that overexpression of miR-125a inhibits growth, invasion, and EMT of CC cells both *in vitro* and *in vivo* by downregulating STAT3 expression. Furthermore, miR-125a induces G2/M cell cycle arrest and inhibits several G2/M checkpoint proteins, suggesting its potential as a biomarker and therapeutic target in CC (Fan et al., 2015). Similarly, a study employed microarray analysis to identify molecular alterations in CC cells treated with TGFβ1 and observed a significant downregulation of miR-374c-5p compared to parental cell lines. Subsequent experiments revealed that ectopic expression of miR-374c-5p suppressed invasion and migration of TGFβ1-treated CC cells, while its knockdown enhanced these processes in parental cell lines. Mechanistically, miR-374c-5p targeted the 3′-UTR of FOXC1, resulting in decreased FOXC1 expression and subsequent suppression of snails. Clinically, low miR-374c-5p expression correlated with poor patient survival and increased lymph node metastasis in CC samples (Huang et al., 2017). Moreover, miR-374c-5p levels negatively correlated with FOXC1 expression, which was elevated in cervical cancers with lymph node metastasis. These findings underscore the pivotal role of miR-374c-5p in regulating CC metastasis via FOXC1 targeting (Huang et al., 2017). Additionally, the change in miR-499a expression either upregulation or downregulation was also associated with change on CC proliferation, formation, progression, migration, and invasion. The increased expression of miR-499a was significantly linked with cell progression. Further, experimentation revealed that the sex-determining region Y box was directly associated with a target of miR-499a. miR-499a-induced SOX6 downregulation mediated the oncogenic effects of miR-499a in CC (Chen Y. et al., 2020). Recently, a study was performed to identify the role of miR-218 and revealed that the overexpression of miR-218 plays a crucial role in tumour metastasis and reduces the proliferation in human CC cell lines HeLa and induces cell apoptosis via AKT-mTOR signalling pathway. Furthermore, miR-218 increased chemosensitivity to cisplatin *in vitro*. This intricate interplay underscores miRNAs’ pivotal role in the pathological development of CC. Regulation of EMT revealed that miR-663b can directly target the 3′UTR of monoacylglycerol acyltransferase 3 (MGAT3) and can participate in the EMT regulatory process (Li et al., 2012).

### 3.5 MiRNAs affect angiogenesis during cervical cancer progression

miRNAs play a significant role in regulating angiogenesis during CC progression. In the context of CC, exosome miRNAs, such as miR-221-3p, have been shown to promote angiogenesis by targeting specific genes involved in this process (Xu et al., 2023). Exosomal miRNAs are secreted by CC cells and taken up by microvascular endothelial cells (MVECs). These exosomal miRNAs can modulate gene expression in MVECs, leading to changes in angiogenic processes (Aslan et al., 2019). Additionally, mir-221-3p downregulates Mitogen-Activated Protein Kinase 10 (MAPK10) expression, which in turn affects downstream factors such as Cellular-Fibrosarcoma Oncogene (c-FOS), Cellular-Jun Proto-Oncogene (c-JUN), Jun B Proto-Oncogene (JUNB), and Vascular Endothelial Growth Factor (VEGF). This cascade of events enhances the abilities of migration, invasion, and angiogenesis in CC cells (Zhang L. et al., 2019). Specifically, miR-205 has been identified as a key player in inhibiting angiogenesis in CC through various mechanisms. Downregulation of miR-205 has been shown to promote angiogenesis by activating the Akt signalling pathway via Tumour Suppressor in Lung Cancer 1 (TSLC1) upregulation. Additionally, miR-205 has been reported to inhibit proliferation, invasion, migration, and angiogenesis in CC by targeting TSLC1 (Zhang F. et al., 2019).

Furthermore, other miRNAs such as miR-187 have also been implicated in inhibiting the growth of CC cells by targeting FGF9. These findings highlight the intricate regulatory roles of miRNAs in modulating angiogenesis and tumour progression in CC (Liang et al., 2023). Moreover, by targeting the hepatocyte growth factor-regulated tyrosine kinase substrate (HGS) mRNA, miR-296 significantly contributes to angiogenesis by lowering HGS levels and slowing down the degradation of growth factor receptors Vascular Endothelial Growth Factor Receptor 2 (VEGFR2) and Platelet-Derived Growth Factor Receptor (PDGFR) beta (Würdinger et al., 2008). A study investigated that miR-129-5p affects cell angiogenesis, invasion, and migration by targeting the Zinc Finger Protein of the Cerebellum 2 (ZIC2) via the Hedgehog signalling pathway in CC. Tissues from 87 CC patients were analysed for miR-129-5p levels, ZIC2 mRNA and protein levels, and Hedgehog pathway components. *In vitro* assays assessed angiogenesis, invasion, and migration, while *in vivo* tumour formation in nude mice analysed angiogenesis and tumour growth (Wang YF. et al., 2018). Upregulating miR-129-5p decreased ZIC2, Shh, Gli1, Gli2, Chemokine (C-X-C Motif) Ligand 1 (CXCL1), VEGF, and Ang2 levels, inhibiting angiogenesis, migration, and invasion in CC cells. Similarly, nude mice showed inhibited tumour growth and angiogenesis (Wang YF. et al., 2018).

Furthermore, a research looked at the involvement of cervical squamous cell carcinoma (CSCC) cell-secreted exosomal miR-221-3p in tumour angiogenesis. Clinical specimens revealed a strong connection between miR-221-3p expression and microvascular density in CSCC. Experiments with CSCC cell lines and miR-221-3p modification verified its accumulation in CSCC exosomes and transferred to human umbilical vein endothelial cells (HUVECs) (Wu et al., 2019). Functional experiments have shown that CSCC exosomal miR-221-3p stimulates angiogenesis *in vitro* and tumour development *in vivo*. Bioinformatic prediction and experimental confirmation revealed thrombospondin-2 (THBS2) as a direct miR-221-3p target, and THBS2 overexpression in HUVECs inhibited miR-221-3p′s angiogenic impact. These findings indicate that CSCC-derived exosomal miR-221-3p might be used as a diagnostic biomarker and therapeutic target for CSCC progression (Wu et al., 2019). Additionally, regulation of angiogenesis observed that upregulation of miR-129–5p inhibits CC cell growth and angiogenesis in naked mice via suppression of the Hedgehog signalling pathway and negative targeting of ZIC2 (Xu et al., 2022).

## 4 Role of miRNAs in cervical cancer resistance to therapeutic regimens

MiRNAs have been shown to play a crucial role in CC resistance to therapeutic regimens. Studies have revealed that dysregulation of specific miRNAs can contribute to the development of resistance to promising therapies such as chemotherapy, immunotherapy, hormonal therapy and radiation therapy in CC patients. These miRNAs function by modulating the expression of target genes involved in drug efflux, DNA repair, and apoptotic pathways, thereby conferring resistance to treatment (Geretto et al., 2017). Furthermore, the dysregulation of miRNAs has been associated with the resistance of CC cells to targeted therapies, such as inhibitors of the epidermal growth factor receptor (EGFR) and VEGF. Research has also indicated that miRNAs can serve as potential biomarkers for predicting the response of CC patients to specific therapeutic interventions. Identifying the expression patterns of these miRNAs in CC tissues may offer valuable insights into the likelihood of treatment success and aid in the personalized management of patients (Wang et al., 2019). Drug resistance poses a formidable obstacle to successful cancer chemotherapy, significantly impacting patient outcomes. Indeed, more than 90% of cancer-related deaths are attributed to the development of drug resistance, underscoring the urgent need for innovative strategies to overcome this challenge (Vasan et al., 2019). Moreover, ongoing investigations are exploring the potential of utilizing miRNA-based therapeutics to overcome resistance mechanisms in CC. By targeting specific dysregulated miRNAs, researchers aim to sensitize cancer cells to conventional therapies and enhance treatment efficacy (Si et al., 2019). The evolving understanding of miRNAs and their intricate involvement in CC resistance underscores the promising prospects for integrating miRNA-based approaches into the comprehensive management of this disease. Continued research in this field holds great potential for advancing the development of innovative strategies to combat therapeutic resistance and improve outcomes for CC patients (Si et al., 2019). Numerous studies underscore the intricate role of miRNAs in orchestrating drug resistance in tumour cells. These miRNAs exert their influence by targeting genes associated with drug resistance or by modulating crucial cellular processes (Si et al., 2019). Notably, a single miRNA can target multiple genes, and its regulatory impact is often tissue-specific, underscoring the complexity of miRNA-mediated drug resistance mechanisms. Additionally (Table 2), offers a thorough summary of the miRNAs that are predominantly responsible for influencing therapeutic treatments in cancer patients. These miRNAs specifically target genes and proteins, providing insight into their complex functions in the mechanisms behind treatment resistance. But other miRNAs also show promise for advancing therapeutic approaches, underscoring their complex influence on CC therapy.

**TABLE 2:** miRNAs implicated in therapeutic regimens of cervical cancer.

miRNA	Effect on treatments	Target genes/proteins	*In vitro*/ In silico/*In vivo*	Reference
miR-499a, miR-181a	• lead to chemoresistance to CDDP	SOX6 and PRKCD	*In vitro*	Masadah et al. (2021)
MiR-6893	• CDDP resistance through autophagy signalling in cancer cells	EMT	*In vitro*	Chen et al. (2019)
miR375	• develop resistance to paclitaxel and promotes EMT	E-cadherin	*In vitro* and *in vivo*	Shen et al. (2014)
miR-21	• chemoresistance to CDDP and paclitaxel in CC cell lines and tissues	SMAD7, Bcl-2, survivin, c-myc, Bax, and PDCD4	*In vitro*	Du et al. (2017)
miR-20a	• restore FBXL5 and BTG3 expression and overcome chemoresistance	SMAD7, Bcl-2, survivin, c-myc, Bax, and PDCD4	*In vitro*	Xiong et al. (2017)
miR-25-3p	• enhanced EMT, migration, and invasion capacities in Caski and Hela cells, as well as resistance to chemotherapy	Sema4c and snail expression	*In vitro*	Song and Li (2017)
miR-155	• greater chemosensitivity to CDDP	TP53, SMAD2 and CCND1	*In vitro*	Bayraktar and Van Roosbroeck (2018)
miR-218	• tumour development	cyclin D1 and CDK4	*In vitro* and *In vivo*	Li et al. (2022)
miR-181a	• radio-resistant CC specimens and cell lines	pro-apoptotic PRKCD gene and protein	*In vitro* and *In vivo*	Ke et al. (2013)
miR-125a	• sensitized CC cells to radiation therapy	p21 (CDKN1A)	*In vitro*	Pedroza-Torres et al. (2018)
MiR-15a-3p	• exposure to radiation • sensitivity to radiation therapy by targeting	TPD52	*In vitro* and *In vivo*	Wu et al. (2018)
miR-4429	• radio-resistant CC cells	RAD51 recombinase (RAD51)	*In vitro* and In silico	Sun et al. (2020)
miR-130a-3p	• contributes to tumour progression	ERα and AR	*In vitro* and *In vivo*	Fan et al. (2021)
miR-107-5p	• promote tumour proliferation and invasion	ERα	*In vitro* and *In vivo*	Kaur and Khatik (2020)
miR-204-5p	• control proliferation and invasion of endometrial carcinoma cells	TrkB-STAT3-	*In vitro*, *In vivo* and In silico	Bao et al. (2013)
miR-138, miR-210, and miR-744	• enhancing the effectiveness of chemotherapy w	ACA and cisplatin	*In vitro*	Shen et al. (2020)
miR-214	• increase sensitivity to cisplatin	Bcl2l2	*In vitro*	Wang et al. (2013)
miR-218	• enhance sensitivity to cisplatin	AKT-mTOR	*In vitro*	Li et al. (2012)
miRNA-21	• regulating T cell function and immune response	PD-1 and PD-L1, PTEN	*In vitro*	Deng et al. (2022)
miR-200	• modulate the response to immunotherapy	EMT	*In vitro* and *In vivo*	Klicka et al. (2022)
miR-155	• modulate the response to immunotherapy	T cells and dendritic cells	*In vitro* and *In vivo*	Kalkusova et al. (2022)

### 4.1 Chemotherapy

Resistance to chemotherapeutic medicines is a significant challenge for cancer therapy. Aberrant miRNA expression is linked to chemoresistance, with other variables including reduced drug absorption, enhanced DNA damage repair, apoptotic inactivation, EMT activation, and epigenetic modifications. However some miRNAs play a dual role in both contributing to chemoresistance and aiding in its overcoming (Karimi and Mollaei, 2021). Recent research illustrates the role of miRNAs in chemoresistance and chemosensitivity in CC. Chemoresistance cell lines, created by increasing chemotherapeutic drug concentrations, are extensively used to examine the function of miRNAs in chemoresistance (Masadah et al., 2021). According to the findings, overexpression of miR-499a and miR-181a in CC cells can lead to chemoresistance to cisplatin (CDDP) by targeting SRY-Box Transcription Factor 6 (SOX6) and Protein Kinase C Delta (PRKCD) (Ke et al., 2013; Chen Y. et al., 2020). Moreover, the study revealed that inhibiting the expression of miR-499a and miR-181a heightened the chemosensitivity to CDDP (Ke et al., 2013). Conversely, previous research has highlighted the role of SOX9 in activating miR130a expression by binding to its promoter (Feng et al., 2018). This activation, in turn, fosters CDDP chemoresistance by downregulating the expression of copper transporter protein 1 (CTR1), a downstream target of miR-130a. The investigation further elucidated the involvement of the SOX9/miR-130a/CTR1 axis in mediating chemoresistance to CDDP in CC (Feng et al., 2018). Moreover, it has been observed that hsc-circ-0023404 suppresses autophagy-induced apoptosis and targets miR-5047, contributing to chemoresistance (Mitra and Elangovan, 2021). Furthermore, cancer cells can develop chemoresistance by triggering EMT. Regulating EMT might help overcome chemoresistance. CC cells develop resistance to paclitaxel by overexpressing miR375, which promotes EMT by targeting E-cadherin (Banno et al., 2014). Overexpression of oncogenic miRNAs, such as miR-20a and miR-21, led to chemoresistance to CDDP and paclitaxel in CC cell lines and tissues (Xiong et al., 2017). The interaction of SMAD7, Bcl-2, survivin, c-myc, Bax, and PDCD4 with miR-21 has been linked to CDDP and paclitaxel resistance in CC cells (Mitra and Elangovan, 2021). Furthermore, depleting or silencing miR-20a in inhibitor of apoptosis-stimulating protein of p53 (iASPP) could potentially restore the expression of F-box and leucine-rich repeat protein 5 (FBXL5) and B-cell translocation gene 3 (BTG3), thereby overcoming chemoresistance (Xiong et al., 2017). Moreover, it was found that downregulation of miR-25-3p is linked to enhanced EMT, migration, and invasion capacities in Caski and Hela cells, as well as resistance to chemotherapy. miR25-3p-mimic therapy can increase chemosensitivity by reducing Sema4c and snail expression and enhancing E-cadherin expression (Song and Li, 2017). Similarly, Overexpression of miR-155 inhibits cell growth and increases chemosensitivity to CDDP via targeting EMT. Overexpressing miR-155 boosted TP53 expression while inhibiting SMAD2 and CCND1, leading to greater chemosensitivity to CDDP (Kalkusova et al., 2022). Lower expression of miR-125a, miR-144, miR-218, miR-506, and miR-1284 was linked to poor prognosis and chemoresistance in CC cells. Inhibiting miR-218 expression was linked to tumour development. MiR-218 mimics increased miR-218 levels and sensitised CC cells to carboplatin by inhibiting cyclin D1 and CDK4 activity, resulting in decreased tumour development and weight (Dong et al., 2014).

### 4.2 Radiotherapy

The correlation between miRNAs and resistance mechanisms is a substantial barrier to improving radiation efficacy CC treatment. Recently several studies have demonstrated the aberrant expression of numerous miRNAs linked to the resistance in radiotherapy in CC treatment (Lizano et al., 2024). A recent *in vitro* and *in vivo* study has unveiled a significant upregulation of miR-181a in radio-resistant CC specimens and cell lines compared to their radio-sensitive counterparts. This heightened expression of miR-181a correlates with decreased sensitivity to radiation treatment, shedding light on a potential mechanism underlying treatment resistance in CC. Additionally, miR-181a promotes resistance by targeting the pro-apoptotic Protein Kinase C Delta (PRKCD) gene. By binding to the 3′UTR of the PRKCD gene, miR-181a lowers the production of the PRKCD protein, which is implicated in apoptosis (Ke et al., 2013). In 2013 a substantial study revealed the differential expression of twenty miRNAs exhibited a consistent pattern of alteration, with 14 miRNAs overexpressed and 6 suppressed in all three radioresistant CC cell variants compared to controls (Zhang et al., 2013). Notably, a miRNA signature comprising 4 miRNAs (miR-630, miR-1246, miR-1290, and miR-3138) displayed over 5-fold increases in radioresistant cells. Further analysis demonstrated that these four miRNAs could be upregulated in CC cells by radiation treatment in both time-dependent and dose-dependent manners. Ectopic expression of these four miRNAs dramatically increased the survival proportion of irradiated CC cells. Additionally, inhibition of miR-630, one of the specific signature miRNAs, could reverse the radio resistance of CC cells (Zhang et al., 2013). Moreover, a study demonstrated that miR-125a was downregulated in patients with CC who did not respond to standard treatment. Radioresistant CC cell lines (SiHa, CaSki, and HeLa) also exhibited low levels of miR-125a compared to sensitive cell lines. miR-125a regulates the expression of p21 (CDKN1A) in CC cells. The overexpression of miR-125a sensitized CC cells to radiation therapy through the downregulation of p21. The p21 protein was found to be overexpressed in radioresistant cell lines, confirming previous studies (Pedroza-Torres et al., 2018). Furthermore, MiR-15a-3p exhibited downregulation in both CC tissues and cell lines. However, its expression significantly increased upon exposure to radiation. Remarkably, overexpression of miR-15a-3p demonstrated an inhibitory effect on cell proliferation and facilitated apoptosis in radiation-exposed cells. Moreover, tumour Protein D52 (TPD52) emerged as a direct target of miR-15a-3p. Inhibition of TPD52 led to suppressed cell proliferation and induced apoptosis. Notably, tumour xenograft experiments underscored that overexpression of miR-15a-3p heightened sensitivity to radiation therapy by targeting TPD52, highlighting its potential as a therapeutic target for enhancing treatment efficacy in CC (Wu et al., 2018). Moreover, the study investigated the role of miR-4429 in CC cell radio-sensitivity. Initially, the downregulation of miR-4429 in CC cells was validated. Crucially, its association with radio resistance was confirmed by observing its decreased expression in radioresistant CC cells. Gain- and loss-of-function assays demonstrated that miR-4429 sensitized CC cells to irradiation (Sun et al., 2020). Bioinformatics methods were used to identify RAD51 recombinase (RAD51) as a miR-4429 target. RAD51 is essential for DNA damage repair and has been linked to cell radioresistance in a variety of malignancies, including CC. Luciferase reporter tests verified the interaction between miR-4429 and RAD51. Rescue tests showed that miR-4429 increased CC cell radiosensitivity via RAD51. This work indicates miR-4429 as a possible therapeutic target for increasing the radiosensitivity of CC cells by inhibiting RAD51 (Sun et al., 2020).

### 4.3 Hormonal therapy

MiRNAs can target and regulate the expression of hormone receptors such as estrogen receptor α (ERα) and androgen receptor (AR). By modulating the levels of these receptors, miRNAs can influence the response to hormonal therapy in CC (Klinge, 2009). MiRNAs can target key components of hormone signalling pathways, affecting the sensitivity of cancer cells to hormonal therapy. For example, miR-130a has been shown to promote. CC cell proliferation and invasion by targeting ERα and AR (Deng et al., 2022). Specific miRNAs can serve as potential therapeutic targets in CC treatment. For instance, miR-130a-3p has been identified as a promising candidate target for the treatment of cervical cancer, as it contributes to tumour progression by suppressing ERα and AR. Moreover, miR-107-5p has been shown to promote tumour proliferation and invasion by targeting ERα in endometrial carcinoma. It may also play a role in regulating hormone receptor expression in CC (Bao et al., 2019). Similarly, a study demonstrated that miR-130a-3p promotes cell proliferation and invasion by targeting ERα and AR in CC. It could potentially influence the response to hormonal therapy in CC patients (Bao et al., 2013). Moreover, study shows that regulatory circuitry involving TrkB-STAT3-miR-204-5p has been shown to control the proliferation and invasion of endometrial carcinoma cells. This miRNA may have implications for hormonal therapy in gynaecological cancers (Bao et al., 2013). However, miR-130a In gastric cancer, miR-130a has been reported to promote migration, invasion, and proliferation by targeting Runt-related transcription factor 3 (RUNX3). Its role in regulating hormone receptors and response to hormonal therapy in CC warrants further investigation (Jiang et al., 2015). Altogether, miRNAs have the potential to serve as powerful predictors of response to hormonal therapy also in CC patients.

### 4.4 Immunotherapy

In recent years, there has been growing interest in understanding the role of miRNAs in the context of immunotherapy for cervical cancer. Immunotherapy aims to harness the body’s immune system to target and destroy cancer cells (Kaur and Khatik, 2020). Therefore, some studies have demonstrated the involvement of miRNA in immunotherapy response subjected to cervical cancer. Recently, it has been noted that miR-138, miR-210, and miR-744 have been shown to improve sensitivity to anticancer agents like ACA and CDDP, thereby enhancing the effectiveness of chemotherapy which indirectly enhances the response of the body against CC cells (Phuah et al., 2013). Moreover, by inhibiting Bcl2l2 expression, miR-214 can increase sensitivity to cisplatin. This inhibition accelerates apoptosis and decreases cell growth by upregulating proapoptotic proteins like Bax, caspase-9, caspase-8, and caspase-3 which somewhere triggers the immune response in order to treat CC (Wang et al., 2013). Similarly, Through the AKT-mTOR signalling pathway, miR-218 can enhance sensitivity to CDDP in CC, making the cancer cells more responsive to chemotherapy (Li et al., 2012). Moreover, miRNA-21 has been shown to target immune checkpoint molecules such as Programmed cell Death protein 1 (PD-1) and Programmed Death-Ligand 1 (PD-L1), which are crucial for regulating T cell function and immune response in the tumour environment. Therefore, targeting miRNA-21 in combination with immunotherapies may enhance the efficacy of treatment by reversing immune suppression and promoting antitumour immunity (Peralta-Zaragoza et al., 2016). Whereas, the miR-200 family members (miR-200a, miR-200b, miR-200c, miR-141, and miR-429) are known to regulate EMT, a process involved in cancer metastasis. Emerging evidence suggests that miR-200 family members may modulate the response to immunotherapy in CC by influencing the tumour microenvironment and immune cell infiltration suggests a promising avenue in enhancing the efficacy of immunotherapy to treat CC (Klicka et al., 2022). Additionally, recent studies have suggested that miR-155 may modulate the response to immunotherapy by regulating the function of immune cells, such as T cells and dendritic cells (Kalkusova et al., 2022). However, Further research is needed to properly understand the roles of miRNAs in regulating the response to immunotherapy and to explore their potential as therapeutic targets in CC treatment.

## 5 Interplay between miRNAs and signalling pathways in cervical cancer

CC is a complex disease characterized by the dysregulation of various signaling pathways, and the interplay between miRNAs and these pathways has garnered significant attention in cancer research. miRNAs have been implicated in the regulation of various signaling pathways involved in CC, including the PI3K/Akt, Wnt/β-catenin, JAK/STAT, and Notch pathways. miRNA-mediated regulation can influence crucial processes such as cell proliferation, apoptosis, and metastasis (Manzo-Merino et al., 2014). The PI3K/AKT/mTOR pathway is frequently dysregulated in CC and plays a crucial role in cell proliferation, survival, and metastasis. miRNAs have been implicated in modulating this pathway by targeting key components such as PI3K, AKT, and mTOR (Bahrami et al., 2017).

Similarly, aberrant activation of the Wnt/β-catenin pathway has been implicated in CC development and progression. miRNAs, such as members of the miR-200 family, have been found to suppress the Wnt/β-catenin pathway by targeting regulators such as ZEB1 and ZEB2, thereby inhibiting epithelial-mesenchymal transition (EMT) and metastasis (Zhang et al., 2024). Conversely, miRNAs, such as miR-21 and miR-135a, promote Wnt/β-catenin signaling by targeting negative regulators, such as APC and GSK-3β, leading to enhanced tumor growth and invasiveness. The MAPK signaling pathway is similarly dysregulated and critical for cell proliferation, survival, and differentiation (Lei et al., 2020). miRNAs have been shown to regulate MAPK pathway activity by targeting components including MAPK kinases and phosphatases. For example, miR-9 has been demonstrated to target MAPK1, which regulates the proliferation and migration of CC cells. Additionally, the JAK/STAT pathway is involved in CC progression and immune evasion. miRNAs can influence JAK/STAT signaling by targeting cytokines, receptors, and downstream effectors. Dysregulation of JAK/STAT pathway activation contributes to CC development and progression. Overall, the interplay between miRNAs and signaling pathways in CC is complex and multifaceted (Valle-Mendiola et al., 2023). Understanding the specific miRNA-mediated regulatory networks within signaling pathways can provide valuable insights into the molecular mechanisms underlying CC, paving the way for the development of novel therapeutic strategies.

### 5.1 PI3K/AKT/mTOR signaling pathway

The phosphoinositide-3 kinase (PI3K), AKT, and mammalian target of rapamycin (mTOR) signaling pathway stand as a pivotal player in driving CC progression, its dynamics meticulously influenced by miRNAs. miRNAs are adept at orchestrating gene expression (Yu et al., 2022). The pathway’s dysregulation fuels tumour development, bolstering growth, invasiveness, and metastasis in CC. Within this intricate landscape, numerous miRNAs have emerged as potent regulators of PI3K/AKT/mTOR, either directly engaging pathway components or indirectly modulating its activity by targeting upstream regulators or downstream effectors (Bahrami et al., 2017). Notable examples include miR-21, miR-214, and miR-126, implicated in promoting CC advancement through PI3K/AKT/mTOR pathway activation, contrasting with miR-145, miR-133a, and miR-218, which act as TS by stifling pathway activation. Deeper insights into the nuanced interplay between miRNAs and the PI3K/AKT/mTOR pathway offer promising avenues for therapeutic intervention and the identification of biomarkers essential for navigating CC management effectively (Doghish et al., 2023). Lately, miR-99b directly targets and negatively regulates the expression of mTOR in CC cells. This downregulation of mTOR expression is crucial in inhibiting the PI3K/AKT/mTOR pathway, which is known to play a significant role in cell proliferation and tumour growth. miR-99b overexpression leads to reduced levels of key proteins in the PI3K/AKT/mTOR signalling cascade, including PI3K, AKT, mTOR, and ribosomal protein S6 kinase (p70S6K) (Li YJ. et al., 2019). By targeting these components, miR-99b disrupts the signalling pathway at multiple levels, inhibiting downstream signalling responses that promote cell proliferation, migration, and survival. Through its regulatory effects on the PI3K/AKT/mTOR pathway, miR-99b suppresses cell proliferation, invasion, and migration in CC cells. This inhibition of cellular activities is essential in slowing down the progression of CC and reducing the aggressiveness of the disease (Li YJ. et al., 2019). Similarly, a study demonstrated that miRNA-383 suppresses the PI3K-AKT-MTOR signalling pathway by targeting and down-regulating PARP2. that miR-383 can negatively regulate PARP2, which in turn leads to the inhibition of PARP2 gene expression. This downregulation of PARP2 by miR-383 results in decreased expression of key components of the PI3K-AKT-MTOR pathway, including PI3K, AKT, mTOR, and p70S6K. By targeting PARP2, miR-383 effectively disrupts the signaling cascade of the PI3K-AKT-MTOR pathway, thereby inhibiting its activity, and potentially attenuating CC progression (Teng et al., 2018). Moreover, a study investigated the functional role and molecular mechanism of miR-125 in CC. Then qRT-PCR was employed to detect miR-125 and VEGF mRNA expression, and western blot analysis to assess protein levels of various markers including VEGF, E-cadherin, N-cadherin, vimentin, AKT, p-AKT, PI3K, p-PI3K, and MTT. Further, transwell assays were utilized to evaluate CC cell progression, including cell viability, migration, and invasion. Consequently, revealed that miR-125 was downregulated while VEGF was upregulated in both CC tissues and cell lines CaSki and SiHa. MiR-125 was found to inhibit proliferation, invasion, and migration by targeting VEGF in cervical cancer. Additionally, miR-125 negatively regulated VEGF expression in CC tissues. Furthermore, demonstrated that miR-520d-5p inhibited the activation of the PI3K/AKT signaling pathway (Fu et al., 2020).

### 5.2 Wnt/b-catenin signaling pathway

The interplay between miRNAs and the Wnt/β-catenin pathway is pivotal in CC progression. miRNAs, regulate gene expression post-transcriptionally, impacting the activity of the Wnt/β-catenin pathway, crucial for cancer development. Various miRNAs directly target Wnt/β-catenin components, exerting dual effects (Peng et al., 2017). For instance, miR-200 family members suppress Wnt/β-catenin signaling, inhibiting EMT and metastasis (Su et al., 2012). Conversely, miR-21 and miR-135a enhance Wnt/β-catenin activation, promoting tumour growth (Gebrie, 2022). Moreover, the pathway reciprocally influences miRNA expression, shaping CC pathogenesis. Wnt ligands are pivotal signaling molecules that initiate the Wnt/β-catenin pathway upon binding to membrane protein receptors (Yang et al., 2018). This interaction triggers a cascade of events, culminating in the accumulation of β-catenin within the cell. Importantly, β-catenin is shielded from phosphorylation by GSK-3β, a crucial step that prevents its degradation by the intracytoplasmic damage complex (Yang et al., 2018). Consequently, non-phosphorylated β-catenin evades ubiquitination and destruction, allowing it to amass in the cytoplasm before translocating into the nucleus (Yang et al., 2018). Once in the nucleus, β-catenin partners with TCF/LEF transcription factors, activating downstream targets such as the cellular myelocytomatosis viral oncogene (c-Myc) (Voronkov and Krauss, 2013). This activation cascade fuels cancer cell proliferation and differentiation, underscoring the pivotal role of the β-catenin-TCF/LEF complex in driving the Wnt/β-catenin signaling pathway (Voronkov and Krauss, 2013). *In vivo*, FAM201A promotes cell survival, migration, and invasion in CC showing that high expression of FAM201A can upregulate FLOT1 expression by sponging of miR-1271-5p. This stimulates the Wnt/β-catenin pathway, promoting CC development and metastasis. AXIN2, a miR-205-5p target gene, has been shown to inhibit Wnt/β-catenin pathway activity. DKK1 and β-catenin are indicators for the Wnt/β-catenin pathway. Low expression of HNRN-PU-AS1 and high expression of miR-205-5p can boost β-catenin production while inhibiting DDK1 expression, activating the Wnt/β-catenin pathway. High expression of AXIN2 suppresses the Wnt/β-catenin pathway, decreasing cell proliferation and inducing apoptosis in CC (Wang et al., 2022).

### 5.3 MAPK signaling pathway

The Mitogen-Activated Protein Kinase (MAPK) signaling pathway is a critical intracellular signaling cascade involved in regulating various cellular processes, including proliferation, differentiation, and apoptosis (Shao et al., 2018). Dysregulation of the MAPK pathway has been implicated in the pathogenesis of CC, contributing to tumour initiation, progression, and metastasis. In recent years, miRNAs have emerged as key regulators of the MAPK pathway in CC (Asl et al., 2021). These small non-coding RNAs modulate gene expression post-transcriptionally, affecting multiple components of the MAPK pathway. Certain miRNAs have been identified to either promote or suppress MAPK pathway activity by targeting key signalling molecules such as Ras, Raf, MEK, and ERK (Asl et al., 2021). For instance, miR-21 has been shown to enhance MAPK pathway activation by targeting negative regulators, thereby promoting CC cell proliferation and invasion (Gebrie, 2022). Conversely, miR-143 acts as a tumour suppressor by inhibiting the MAPK pathway, thereby suppressing CC progression. Understanding the intricate interplay between miRNAs and the MAPK pathway in CC provides valuable insights into the molecular mechanisms underlying tumorigenesis (Zhao et al., 2017). As a result, a study utilized qRTPCR to analyze the expression of MiR-338-3p and MACC1 in CC and investigated the effects of miR-338-3p and MACC1 on cell growth. A luciferase reporter assay was used to confirm the target gene of miR-338-3p in CC cells. Hence, identified MACC1 as a functional downstream target of miR-338-3p. Overexpressing miR-338-3p lowered MACC1 expression in CC cells, potentially inhibiting CC (Hua et al., 2017). Moreover, researchers investigated the role of miR-329-3p in CC. They found that miR-329-3p expression was reduced in CC tissues and cell lines and correlated with tumour grade, stage, and lymph node metastasis. Upregulation of miR-329-3p inhibited cell proliferation, migration, and invasion (Li et al., 2017). The researchers identified MAPK1 as a direct target gene of miR-329-3p, which was upregulated in CC tissues and inversely correlated with miR-329-3p expression. Silencing MAPK1 mimicked the effects of miR-329-3p overexpression, while restoring MAPK1 expression reversed these effects. These findings suggest that miR-329-3p acts as a tumour suppressor in CC by targeting MAPK1, indicating its potential as a therapeutic target for this disease (Li et al., 2017). Downregulation of miR-99a and miR-125b-2 in CC leads to TRIB2, HOXA1, and mTOR overexpression (Gao et al., 2018). Research indicates that the oncogene TRIB2 is elevated in verities of malignancies and may influence the selectivity of MAPK activation. Similarly, the transcription factor HOXA1 boosts cancer indicators by activating MAPK signalling (Granados-López et al., 2017). Furthermore, an investigation assessed expression levels of HOTAIR and miR-23b in CC samples using real-time PCR. Results indicated elevated HOTAIR and reduced miR-23b expression in cancerous tissues and cell lines (Li et al., 2018). Knockdown of HOTAIR led to apoptosis promotion, as well as inhibition of cell proliferation and invasion both *in vitro* and *in vivo*. Additionally, HOTAIR was found to potentially competitively bind miR-23b, thereby indirectly modulating MAPK1 expression (Li et al., 2018). These findings unveil a novel oncogenic pathway involving HOTAIR in CC, offering insights into its potential prognostic and therapeutic implications.

### 5.4 JAK/STAT signaling pathway

The JAK/STAT signalling pathway, a crucial player in regulating cell growth, survival, and differentiation, is tragically misconstrued in the context of CC (Luo and Balko, 2019). The Janus kinase (JAK) family initiates a cascade in response to various cytokines and growth factors. This activates STAT proteins, which translocate to the nucleus and orchestrate changes in gene expression. However, in CC, this complementary movement transforms into a turbulent result (Dutta and Li, 2013). HPV oncoproteins, particularly E6 and E7, disrupt this pathway by directly activating JAKs or by modifying STAT proteins. This leads to persistent activation of STAT3, a key player in promoting cell proliferation, migration, and resistance to apoptosis. Additionally, chronic inflammation, often associated with HPV infection, fuels this pathway via cytokines like interleukin‐6 (IL-6) (Gutiérrez-Hoya and Soto-Cruz, 2020). This dysregulated JAK/STAT signaling fosters a conducive environment to tumorigenesis and contributes to aggressive cancer phenotypes. Specific JAK inhibitors and STAT3 antagonists are being explored, aiming to silence oncogenic music and restore cellular symphony. Challenges such as potential off-target effects and complex interactions with other pathways exist; unraveling the JAK/STAT story in CC offers a beacon of hope for novel and effective therapeutic interventions (Valle-Mendiola et al., 2023). However, a study demonstrated the downregulation of miR‐9 in cervical adenocarcinoma due to frequent hypermethylation, exerting a tumor suppressor role by targeting various genes, including IL‐6. Hypermethylation of miR‐9 precursor promoters was observed in cervical adenocarcinoma tissues, and demethylation treatment increased mature miR‐9 expression in HeLa cells. Some assays revealed CKAP2, HSPC159, IL‐6, and TC10 as novel direct target genes of miR‐9, with pathway analysis indicating their involvement in the Jak/STAT3 pathway downstream of IL‐6. Ectopic miR‐9 expression inhibited Jak/STAT3 signalling activity, which was partially reversed by exogenous IL‐6. Overall, miR‐9 has the potential to suppress tumors in cervical adenocarcinoma and suggests repression of tumorigenesis through inhibition of the IL‐6/Jak/STAT3 pathway (Zhang et al., 2016). Moreover, miR-126 functions as a tumor suppressor in CC cells *in vitro*, which inhibits proliferation, migration, and invasion by suppressing MMP2 and MMP9 expression and inactivating the JAK2/STAT3 signalling pathway by targeting ZEB1, suggesting that miR-126 may be a novel potential target for the diagnosis and treatment of patients with CC (Xu et al., 2019).

## 6 MicroRNAs as diagnostic and prognostic biomarkers for cervical cancer

miRNAs have become prominent biomarkers for assessing the diagnosis and prognosis of CC, attributable to their aberrant expression profiles in cancerous tissues relative to healthy tissues (Banno et al., 2014). Numerous studies have shown the dysregulation of specific miRNAs in CC tissues and their potential role in disease progression. These small non-coding RNAs can be easily detected in body fluids such as blood and cervical secretions, making them attractive candidates for non-invasive diagnostic tests. Several specific miRNAs have been identified as potential biomarkers for CC. For instance, miR-21, miR-182, miR-183, miR-214, and miR-224 have been consistently upregulated in CC cells and tissues, while miR-150, miR-200b, miR-636, miR-205, and miR-187 are commonly downregulated (Mitra and Elangovan, 2021; Shademan et al., 2023). Detecting dysregulated miRNAs in patient samples shows potential for creating non-invasive CC screening tools, especially in resource-limited settings. Additionally, miRNAs have prognostic value in CC, correlating with clinical parameters like tumor stage, lymph node metastasis, and patient survival (Chen S. et al., 2020). High expression levels of certain miRNAs, such as miR-224 and miR-182, have been associated with advanced tumour stage, lymph node metastasis, and poor prognosis in CC patients. Conversely, low expression levels of tumour-suppressive miRNAs like miR-150, miR-200b, miR-636, miR-205, and miR-187 have been linked to aggressive tumour behaviour, induce cell apoptosis and reduced overall survival rates (Sharma and Gupta, 2020). These findings suggest that miRNA expression profiles could serve as reliable prognostic indicators to guide treatment decisions and improve patient outcomes. In addition to their diagnostic and prognostic value, miRNAs show potential as therapeutic targets for CC. Modulating miRNA expression levels using miRNA mimics has shown potential in preclinical studies to inhibit tumour growth, metastasis, and enhance chemosensitivity in CC models (Di Fiore et al., 2022). Recently, a bioinformatic approach has investigated that that miR-21 may function as a highly sensitive and specific marker for the diagnosis of CC (Deng et al., 2022). Additionally, a study showed miRNA-21 is an oncogenic miRNA molecule playing a key role in the development and progression of cervical malignancy. Whereas it has good diagnostic accuracy as well. In addition, the upregulation of miRNA-21 could predict a worse outcome in terms of prognosis in CC patients (Deng et al., 2022). Moreover, miR-885-5p expression was decreased in CC, and downregulation of miR-885-5p promoted the progression of CC cells has been elucidated via an investigation and concluded that miR-885-5p may be an independent prognostic predictor and therapeutic target for treating CC (Zu et al., 2022). A study examined the expression of certain miRNAs in various samples from women with cervical precancer and cancer to explore their potential as non-invasive biomarkers for diagnosing and prognosing CC. They assessed the levels of three oncomiRs (miR-21, miR-199a, and miR-155-5p) and three tumor suppressor (TS) miRNAs (miR-34a, miR-145, and miR-218) using qRT-PCR and correlated their expression with clinicopathological parameters and survival outcomes. The findings revealed significant overexpression of oncomiRs and downregulation of TS miRNAs (Aftab et al., 2021). A combination of miR-145-5p, miR-218-5p, and miR-34a-5p in urine achieved high sensitivity and specificity in distinguishing precancer and cancer patients from healthy controls, with a correlation to serum and tumor tissue expression. Additionally, miR-34a-5p and miR-218-5p were identified as independent prognostic factors for overall survival in CC patients. The findings suggest that evaluating specific miRNA expression in non-invasive urine samples could serve as a reliable biomarker for early detection and prognosis of CC (Garofalo et al., 2014). However, further research is needed to validate the efficacy and safety of miRNA-based therapies for clinical use in CC patients.

## 7 Therapeutic interventions of miRNAs in cervical cancer

CC is a major health issue worldwide, necessitating the development of new treatment techniques to enhance patient outcomes. Recently, miRNA-based medicines have emerged as potential treatments for CC. Nucleic acid-based therapies, such as miRNA mimic therapy, anti-miRNA therapy, and their combination with radiotherapy, hold significant promise in medicine (Garofalo et al., 2014). These approaches exploit the unique regulatory roles of miRNAs in modulating gene expression and cellular pathways. MiRNA mimic therapy involves the use of synthetic miRNA mimics to supplement or enhance the function of specific miRNAs that may be expressed or dysfunctional in certain diseases. Thus, miR-143 is a TS miRNA that is often downregulated in CC. Studies have shown that delivery of synthetic miR-143 mimics can inhibit cell proliferation, induce apoptosis, and suppress tumor growth in CC cell lines (Liu et al., 2018). miR-218 is another TS miRNA that plays a role in CC progression. Studies have demonstrated that the introduction of synthetic miR-218 mimics can inhibit cell migration, invasion, and EMT in CC cells, suggesting a potential therapeutic approach for metastatic CC (Li et al., 2022). Although miR-21 is often associated with oncogenic functions in various cancers, including CC, its role in this context is complex. Some studies have explored the use of synthetic miR-21 mimics to investigate their effects on CC cell proliferation, migration, and invasion, highlighting the potential dual roles of miR-21 in cervical tumorigenesis (Bhattacharjee et al., 2022). Furthermore, miR-181a has been implicated in chemoresistance of cervical squamous cell carcinoma. Research has shown that delivery of synthetic miR-181a mimics can enhance chemosensitivity to cisplatin by targeting specific genes involved in drug resistance pathways, offering a potential strategy to overcome resistance in CC treatment (González-Quintana et al., 2016). Anti-miRNA therapy targets specific miRNAs promoting cancer. In CC, upregulated miR-21 accelerates tumor growth and metastasis. Preclinical studies use anti-miR-21 oligonucleotides to block miR-21, curbing oncogenic pathways, and decreasing cell proliferation and invasion (Toden et al., 2021). miR-155 is another miRNA implicated in CC progression and immune evasion. Research has investigated the use of anti-miR-155 oligonucleotides to block miR-155 function and restore immune responses in the tumor microenvironment, potentially enhancing the antitumor immune response in CC (Bayraktar and Van Roosbroeck, 2018). Furthermore, miR-10b is involved in promoting metastasis and invasiveness of CC. Therefore, studies have explored the use of anti-miR-10b oligonucleotides to inhibit miR-10b expression and to suppress the metastatic properties of CC cells (Menon et al., 2022). miR-34a is a tumor suppressor miRNA that is often downregulated in CC. Interestingly, researchers have investigated the use of anti-miR-34a oligonucleotides to antagonize miR-34a inhibition and restore its tumor-suppressive functions, leading to decreased proliferation and enhanced apoptosis in CC cells (Yin et al., 2023). However, Combining miRNA-based therapies with radiotherapy for the treatment of CC holds promise for enhancing the treatment efficacy and overcoming resistance mechanisms. Consequently, preclinical studies have shown that upregulation of miR-34a in CC cells can sensitize them to radiotherapy by enhancing apoptosis and inhibiting cell proliferation. Combining miR-34a mimics with radiotherapy has demonstrated synergistic effects in reducing tumor growth and improving treatment outcomes in CC models (Yin et al., 2023). Moreover, inhibition of miR-21, which is often upregulated in CC and is associated with radioresistance, has been investigated as a strategy to enhance the efficacy of radiotherapy. Studies have shown that combining anti-miR-21 oligonucleotides with radiotherapy can sensitize CC cells to radiation-induced cell death and overcome radioresistance (Javanmardi et al., 2016). Modulation of miR-155 expression in CC cells has also been explored as a potential approach to enhance the effects of radiotherapy. By using miR-155 mimics or inhibitors in combination with radiotherapy, researchers aim to modulate immune responses, the tumor microenvironment, and radiation sensitivity to improve treatment outcomes in CC (Bayraktar and Van Roosbroeck, 2018). Furthermore, miR-218, a tumor suppressor miRNA that is downregulated in CC, has been studied for its potential role in enhancing the effects of radiotherapy (Banno et al., 2014). These nucleic acid-based therapies represent innovative approaches to precision medicine, offering the potential for targeted and personalized treatment strategies for CC. Researchers are exploring new avenues for improving patient outcomes and advancing the field of therapeutic nucleic acids by harnessing the regulatory functions of miRNAs and combining them with established treatment modalities, such as radiotherapy. However, more detailed investigations are required to validate the efficacy of nucleic acid-based therapies in the management of CC.

## 8 Conclusion

This comprehensive review delineates the intricate roles of miRNAs in the pathogenesis and treatment response of CC. By exerting influence over a myriad of critical processes in CC progression, such as cancer cell proliferation, evasion of apoptosis, cell cycle regulation, invasion, angiogenesis, and metastasis, miRNAs have emerged as key regulators in this formidable disease. Notably, modulation of pivotal molecular pathways, including the PI3K/AKT/mTOR, Wnt/β-catenin, JAK/STAT, and MAPK pathways, underscores their significance in CC biology. Moreover, their involvement in therapy resistance highlights the pressing need for targeted therapeutic strategies against miRNAs in CC pathogenesis along with their potential as diagnostic and prognostic biomarkers. This review also sheds light on therapeutic interventions involving miRNAs in CC, paving the way for future research endeavors aimed at deciphering their precise mechanisms and exploiting their therapeutic potential to combat CC effectively. However, further detailed investigations are warranted to unravel the intricate mechanisms underlying the role of miRNAs as potent players in CC pathogenesis and therapeutic response.
